# Association Between Time to Defibrillation and Survival in Pediatric In-Hospital Cardiac Arrest With a First Documented Shockable Rhythm

**DOI:** 10.1001/jamanetworkopen.2018.2643

**Published:** 2018-09-21

**Authors:** Elizabeth A. Hunt, Jordan M. Duval-Arnould, Melania M. Bembea, Tia Raymond, Aaron Calhoun, Dianne L. Atkins, Robert A. Berg, Vinay M. Nadkarni, Michael Donnino, Lars W. Andersen

**Affiliations:** 1 Division of Health Informatics, Johns Hopkins University School of Medicine, Baltimore, Maryland; 2 Department of Anesthesiology & Critical Care, Johns Hopkins University School of Medicine, Baltimore, Maryland; 3 Department of Pediatrics, Johns Hopkins University School of Medicine, Baltimore, Maryland; 4 Department of Pediatric Cardiology and Pediatric Critical Care, Medical City Children’s Hospital, Dallas, Texas; 5 Department of Pediatrics, University of Louisville School of Medicine, Louisville, Kentucky; 6 Stead Family Department of Pediatrics, Carver College of Medicine, University of Iowa, Iowa City; 7 Department of Anesthesiology, Critical Care, and Pediatrics, University of Pennsylvania, Philadelphia; 8 Department of Medicine, Division of Pulmonary, Critical Care, and Sleep Medicine, Beth Israel Deaconess Medical Center, Boston, Massachusetts; 9 Center for Resuscitation Science, Department of Emergency Medicine, Beth Israel Deaconess Medical Center, Boston, Massachusetts; 10 Research Center for Emergency Medicine, Department of Clinical Medicine, Aarhus University Hospital, Aarhus, Denmark

## Abstract

**Importance:**

Delayed defibrillation (>2 minutes) in adult in-hospital cardiac arrest (IHCA) is associated with worse outcomes. Little is known about the timing and outcomes of defibrillation in pediatric IHCA.

**Objective:**

To determine whether time to first defibrillation attempt in pediatric IHCA with a first documented shockable rhythm is associated with survival to hospital discharge.

**Design, Setting, and Participants:**

In this cohort study, data were obtained from the Get With The Guidelines–Resuscitation national registry between January 1, 2000, and December 31, 2015, and analyses were completed by October 1, 2017. Participants were pediatric patients younger than 18 years with an IHCA and a first documented rhythm of pulseless ventricular tachycardia or ventricular fibrillation and at least 1 defibrillation attempt.

**Exposures:**

Time between loss of pulse and first defibrillation attempt.

**Main Outcomes and Measures:**

The primary outcome was survival to hospital discharge. Secondary outcomes were return of circulation, 24-hour survival, and favorable neurologic outcome at hospital discharge.

**Results:**

Among 477 patients with a pulseless shockable rhythm (median [interquartile range] age, 4 years [3 months to 14 years]; 285 [60%] male), 338 (71%) had a first defibrillation attempt at 2 minutes or less after pulselessness. Children were less likely to be shocked in 2 minutes or less for ward vs intensive care unit IHCAs (48% [11 of 23] vs 72% [268 of 371]; *P* = .01]). Thirty-eight percent (179 patients) survived to hospital discharge. The median (interquartile range) reported time to first defibrillation attempt was 1 minute (0-3 minutes) in both survivors and nonsurvivors. Time to first defibrillation attempt was not associated with survival in unadjusted analysis (risk ratio [RR] per minute increase, 0.96; 95% CI, 0.92-1.01; *P* = .15) or adjusted analysis (RR, 0.99; 95% CI, 0.94-1.06; *P* = .86). There was no difference in survival between those with a first defibrillation attempt in 2 minutes or less vs more than 2 minutes in unadjusted analysis (132 of 338 [39%] vs 47 of 139 [34%]; RR, 0.87; 95% CI, 0.66-1.13; *P* = .29) or multivariable analysis (RR, 0.99; 95% CI, 0.75-1.30; *P* = .93). Time to first defibrillation attempt was also not associated with secondary outcome measures.

**Conclusions and Relevance:**

In contrast to published adult IHCA and pediatric out-of-hospital cardiac arrest data, no significant association was observed between time to first defibrillation attempt in pediatric IHCA with a first documented shockable rhythm and survival to hospital discharge.

## Key Points

**Question:**

In children who have an in-hospital cardiac arrest with a first documented shockable rhythm, is time to first defibrillation attempt associated with survival to hospital discharge?

**Findings:**

In a cohort study from the Get With The Guidelines–Resuscitation national registry of 477 pediatric patients who experienced in-hospital cardiac arrest, time to first defibrillation attempt was not associated with survival. Time to first defibrillation was also not associated with return of circulation, 24-hour survival, or favorable neurologic outcome.

**Meaning:**

In contrast to published adult in-hospital cardiac arrest and pediatric out-of-hospital cardiac arrest data, there was no significant association between time to first defibrillation attempt in pediatric in-hospital cardiac arrests with first documented shockable rhythm and survival to hospital discharge.

## Teaser

This cohort study compares survival to discharge after in-hospital cardiac arrest with a first documented shockable rhythm between children who were treated with defibrillation 2 minutes or less after arrest vs more than 2 minutes after arrest using data from the American Heart Association’s Get With The Guidelines–Resuscitation national registry.

## Introduction

In-hospital cardiac arrest (IHCA) occurs in approximately 6000 children each year in the United States.^1,2,3^ Survival following pediatric IHCA has improved over the last decade, but there is wide variability in processes of care,^4,5,6,7^ and poor outcomes are still common.^8^ This suggests variability in time-sensitive interventions may be a target to improve outcomes.^9,10^

Although many IHCAs in children have a noncardiac origin,^11,12,13,14,15^ 10% to 15% have a first documented rhythm that requires defibrillation.^12,15,16^ Early defibrillation in pediatric cardiac arrest has been recommended since 1977,^17^ and recent guidelines from both the European Resuscitation Council and the American Heart Association recommend defibrillation as soon as possible after the shockable rhythm is recognized.^18,19^ Delayed defibrillation (>2 minutes) in adult IHCA is associated with worse clinical outcomes, with each additional minute of delay resulting in worse survival.^20^ In adults, delayed defibrillation attempts greater than 2 minutes are a national quality measure used by the American Heart Association Get With The Guidelines*–*Resuscitation (GWTG-R) national registry of cardiopulmonary resuscitation (CPR) awards program.^21^ Although pediatric simulation studies reveal delayed defibrillation is common,^6,22^ to our knowledge there are no large population studies reporting the timing of the first defibrillation in pediatric IHCA and whether delayed time to first defibrillation attempt is associated with worse outcomes.

The primary objective of this study was to assess the association between time to first defibrillation attempt in pediatric IHCA with a first documented shockable rhythm and survival to hospital discharge. We hypothesized that delay in first defibrillation attempt after onset of pulseless shockable IHCA would be associated with decreased survival to hospital discharge.

## Methods

### Data Source and Study Population

Data were obtained from the GWTG-R registry, an American Heart Association–sponsored, prospective, quality improvement registry of IHCA in the United States. Additional details about the registry have been described elsewhere.^23^ All data were deidentified. Per the Johns Hopkins institutional review board, this activity is not human subjects research and did not require a submission for review. The study followed the Strengthening the Reporting of Observational Studies in Epidemiology (STROBE) reporting guideline.

Cardiac arrest is defined as pulselessness, or a pulse with inadequate perfusion, requiring chest compressions and/or defibrillation, with a hospital-wide or unit-based emergency response. Patients with prior do-not-resuscitate orders or cardiopulmonary resuscitation events that began outside the hospital were not included.

For this study, inclusion criteria were (1) events occurring from January 1, 2000, to December 31, 2015, for children younger than 18 years who were pulseless and received chest compressions with a first documented pulseless rhythm of pulseless ventricular tachycardia (pVT) or ventricular fibrillation (VF) (ie, a shockable rhythm), and (2) at least 1 defibrillation attempt (shock) provided. No sample size calculation was performed because the sample size was fixed by the size of the cohort, and the dates were chosen to accumulate the largest possible cohort. Patients with a first documented rhythm of pulseless electrical activity or asystole that converted to a shockable rhythm were not included. We excluded events in which the shock was delivered before loss of pulses, events in patients with an automatic implantable cardioverter-defibrillator, and, for the primary analysis, events in which the shock was delivered more than 10 minutes after loss of pulses (as these were few and considered to be either potential recording mistakes or unique clinical situations). For the primary analysis, we excluded subsequent IHCA events within the same patient and events with missing data on the defibrillation attempt, covariates, or survival to hospital discharge.

### Time to First Defibrillation Attempt and Outcomes

Our exposure variable was documented time to first defibrillation attempt, defined as the time interval in minutes from recognition of loss of pulse to the first shock. Shocks were delivered with automated external or manual defibrillators. All times in the GWTG-R registry are collected in whole minutes. As such, a time to first defibrillation of 0 minutes indicates that the defibrillation attempt was performed within the same whole minute as pulses were lost, a time of 1 minute indicates that defibrillation attempt was performed within the next whole minute, and so on.

The primary outcome was survival to hospital discharge. Our secondary outcomes were return of circulation (ROC), 24-hour survival, and favorable neurologic outcome at hospital discharge. We defined ROC as no further need for chest compressions (including initiation of cardiopulmonary bypass or extracorporeal membrane oxygenation) sustained for more than 20 minutes. Neurologic outcome was reported per Utstein guidelines^24^ using the pediatric cerebral performance category (PCPC) score,^25^ in which a PCPC score of 1 indicates no neurologic deficit; 2, mild cerebral disability; 3, moderate cerebral disability; 4, severe cerebral disability; 5, coma or vegetative state; and 6, brain death. A PCPC score of 1 or 2 was considered a favorable neurologic outcome and a PCPC score of 3 to 6 was considered a poor neurologic outcome.^10,25,26^ Sensitivity analyses were performed with different definitions of favorable functional outcome as previously done^27^: (1) a PCPC score of 1 or 2, or no increase from baseline; (2) a PCPC score of 1, 2, or 3; and (3) a PCPC score of 1, 2, or 3, or no increase from baseline.

### Statistical Analysis

For descriptive statistics, categorical variables are presented as counts (frequencies) and continuous variables as means (standard deviations) or medians (interquartile range [IQR]) depending on distribution of the data. We assessed the unadjusted association between time to first shock as a continuous, linear variable in our primary analysis using modified Poisson regression models with robust variance estimates to estimate risk ratios (RRs).^28,29^ To assess the adjusted association between time to first shock and survival to discharge, we applied a multivariable modified Poisson regression model with generalized estimating equations with an exchangeable variance-covariance matrix to account for within-hospital clustering. To create a parsimonious model and avoid overfitting, we first assessed whether included variables were associated with the outcome in unadjusted analysis using a Fisher exact test for categorical variables and a Wilcoxon rank-sum test for continuous variables. All variables associated with the outcome (*P* < .10) were entered into the multivariable model, and modified backward selection was applied. Variables were removed from the model one by one according to the highest *P* value until only variables associated with the outcome (*P* < .05) remained. If removal of a variable resulted in a greater than 10% change in the RR for the association between time to defibrillation and the outcome, the variable was added back into the model. Time to first defibrillation was retained in the model irrespective of the *P* value. All variables in Table 1 were assessed for inclusion in the adjusted model. All variables were chosen a priori based on prior work and clinical reasoning.^30,31,32,33^ To assess whether there was a nonlinear relationship between time to first defibrillation attempt and the primary outcome measure, we added polynomial terms (quadratic and cubic) to the final multivariable model for the primary outcome.

**Table 1.** Characteristics of the Study Population Stratified by Survival Status and Time to Defibrillation Status

Characteristic	No. (%)						
	All Patients (N = 477)	Nonsurvivors (n = 298)	Survivors (n = 179)	*P* Value	≤2 min to Defibrillation (n = 338)	>2 min to Defibrillation (n = 139)	*P* Value
Demographic characteristics							
Sex							
Male	285 (60)	179 (60)	106 (59)	.85	200 (59)	85 (61)	.76
Female	192 (40)	119 (40)	73 (41)		138 (41)	54 (39)	
Age group							
Neonate (<1 mo)	89 (19)	53 (18)	36 (20)	.44	63 (19)	26 (19)	.92
Infant (1 mo to 1 y)	77 (16)	43 (14)	34 (19)		57 (17)	20 (14)	
Child (1-12 y)	161 (34)	103 (35)	58 (32)		114 (34)	47 (34)	
Adolescent (>12 y)	150 (31)	99 (33)	51 (28)		104 (31)	46 (33)	
Illness category							
Medical cardiac	131 (27)	63 (21)	68 (38)	<.001	99 (29)	32 (23)	.19
Medical noncardiac	123 (26)	102 (34)	21 (12)		77 (23)	46 (33)	
Newborn	7 (1)	2 (1)	5 (3)		5 (1)	2 (1)	
Surgical cardiac	138 (29)	63 (21)	75 (42)		102 (30)	36 (26)	
Surgical noncardiac	78 (16)	68 (23)	10 (6)		55 (16)	23 (17)	
Preexisting conditions							
Heart failure this admission	90 (19)	53 (18)	37 (21)	.44	71 (21)	19 (14)	.07
Heart failure prior to this admission	56 (12)	33 (11)	23 (13)	.56	42 (12)	14 (10)	.53
Myocardial infarction failure this admission	13 (3)	7 (2)	6 (3)	.51	10 (3)	3 (2)	.76
Myocardial infarction prior to this admission	5 (1)	2 (1)	3 (2)	.30	5 (1)	0	.33
Hypotension	147 (31)	106 (36)	41 (23)	.004	114 (34)	33 (24)	.04
Respiratory insufficiency	236 (49)	175 (59)	61 (34)	<.001	166 (49)	70 (50)	.84
Renal insufficiency	52 (11)	45 (15)	7 (4)	<.001	32 (9)	20 (14)	.14
Hepatic insufficiency	21 (4)	18 (6)	3 (2)	.02	15 (4)	6 (4)	1.00
Metabolic or electrolyte abnormality	82 (17)	58 (19)	24 (13)	.09	53 (16)	29 (21)	.18
Baseline depression in central nervous system function	56 (12)	49 (16)	7 (4)	<.001	34 (10)	22 (16)	.09
Acute stroke	4 (1)	3 (1)	1 (1)	.60	1 (0)	3 (2)	.08
Acute nonstroke central nervous system event	49 (10)	44 (15)	5 (3)	<.001	34 (10)	15 (11)	.87
Pneumonia	28 (6)	24 (8)	4 (2)	.009	17 (5)	11 (8)	.28
Septicemia	53 (11)	42 (14)	11 (6)	.008	36 (11)	17 (12)	.63
Major trauma	60 (13)	55 (18)	5 (3)	<.001	40 (12)	20 (14)	.45
Metastatic or hematologic malignancy	11 (2)	8 (3)	3 (2)	.48	5 (1)	6 (4)	.09
Location and time of cardiac arrest							
Location							
Critical care area	371 (78)	239 (80)	132 (74)	.001	268 (79)	103 (74)	.03
Emergency department	35 (7)	29 (10)	6 (3)		22 (7)	13 (9)	
Floor with telemetry or step-down unit	7 (1)	3 (1)	4 (2)		5 (1)	2 (1)	
Floor without telemetry	16 (3)	8 (3)	8 (4)		6 (2)	10 (7)	
Other	48 (10)	19 (6)	29 (16)		37 (11)	11 (8)	
Time of week							
Weekday	359 (75)	219 (73)	140 (78)	.25	249 (74)	110 (79)	.24
Weekend	118 (25)	79 (27)	39 (22)		89 (26)	29 (21)	
Time of day							
Daytime	350 (73)	208 (70)	142 (79)	.02	252 (75)	98 (70)	.36
Nighttime	127 (27)	90 (30)	37 (21)		86 (25)	41 (30)	
Characteristic of cardiac arrest							
Witnessed	462 (97)	289 (97)	173 (97)	.84	9 (3)	6 (4)	.39
Monitored	458 (96)	288 (97)	170 (95)	.37	328 (97)	130 (94)	.12
Mechanical ventilation in place	330 (69)	218 (73)	112 (63)	.02	242 (72)	88 (63)	.08
Vasopressors in place	206 (43)	140 (47)	66 (37)	.03	153 (45)	53 (38)	.16
Antiarrhythmic in place	42 (9)	24 (8)	18 (10)	.46	27 (8)	15 (11)	.37
Initial pulseless rhythm							
Pulseless ventricular tachycardia	192 (40)	121 (41)	71 (40)	.84	130 (38)	62 (45)	.22
Ventricular fibrillation	285 (60)	177 (59)	108 (60)		208 (62)	77 (55)	
Time to chest compressions, median (IQR), min,	0	0	0	.71	0	0	.22
Hospital characteristics							
Type of hospital							
Primarily adult	251 (53)	168 (56)	83 (46)	.03	180 (53)	71 (51)	.69
Primarily children	226 (47)	130 (44)	96 (54)		158 (47)	68 (49)	
Teaching status							
Major	352 (74)	210 (70)	142 (79)	.10	247 (73)	105 (76)	.88
Minor	102 (21)	72 (24)	30 (17)		74 (22)	28 (20)	
Nonteaching	23 (5)	16 (5)	7 (4)		17 (5)	6 (4)	
Year of cardiac arrest							
2000-2005	160 (34)	117 (39)	43 (24)	.002	118 (35)	42 (30)	.45
2006-2010	169 (35)	100 (34)	69 (39)		114 (34)	55 (40)	
2011-2016	148 (31)	81 (27)	67 (37)		106 (31)	42 (30)	

Abbreviation: IQR, interquartile range.

A similar approach was used to analyze secondary outcomes (ROC, 24-hour survival, and survival to discharge with favorable neurologic outcome). Results from these multivariable regression models are reported as RRs with 95% confidence intervals for the outcome per minute increase in time to first defibrillation attempt.

We performed the following preplanned sensitivity and subgroup analyses: (1) time to first defibrillation attempt dichotomized into 2 minutes or less and greater than 2 minutes, (2) multiple imputation to account for missing data, and (3) analysis with ROC as the outcome, including subsequent IHCA (ie, recurrent IHCA within the same patient). Additional details are provided in the eAppendix in the Supplement. We also performed several post hoc analyses to address the relationship of time to first defibrillation attempt with outcomes in the following populations: (1) subgroup excluding patients receiving vasopressors, inotropes, or antiarrhythmics at the time of the IHCA, (2) subgroup excluding those receiving chest compressions prior to pulselessness, and (3) subgroup in which the cohort was expanded to include time to first defibrillation attempt up to 20 minutes and including a comparison of time to defibrillation of 2 minutes or less vs more than 12 minutes. We modeled the post hoc analysis of the extremes of time to initial defibrillation attempt (ie, ≤2 minutes vs >12 minutes) per the methods of Herlitz et al^34^ for adult IHCA and Mitani et al^35^ for pediatric out-of-hospital cardiac arrest (OHCA). All analyses were completed by October 1, 2017.

All hypothesis tests were 2-sided with a significance level of *P* < .05. No adjustments were made for multiple testing; thus, secondary analyses should be considered exploratory. Statistical analyses were conducted with SAS statistical software, version 9.4 (SAS Institute).

## Results

### Patient Characteristics

**Figure 1.**
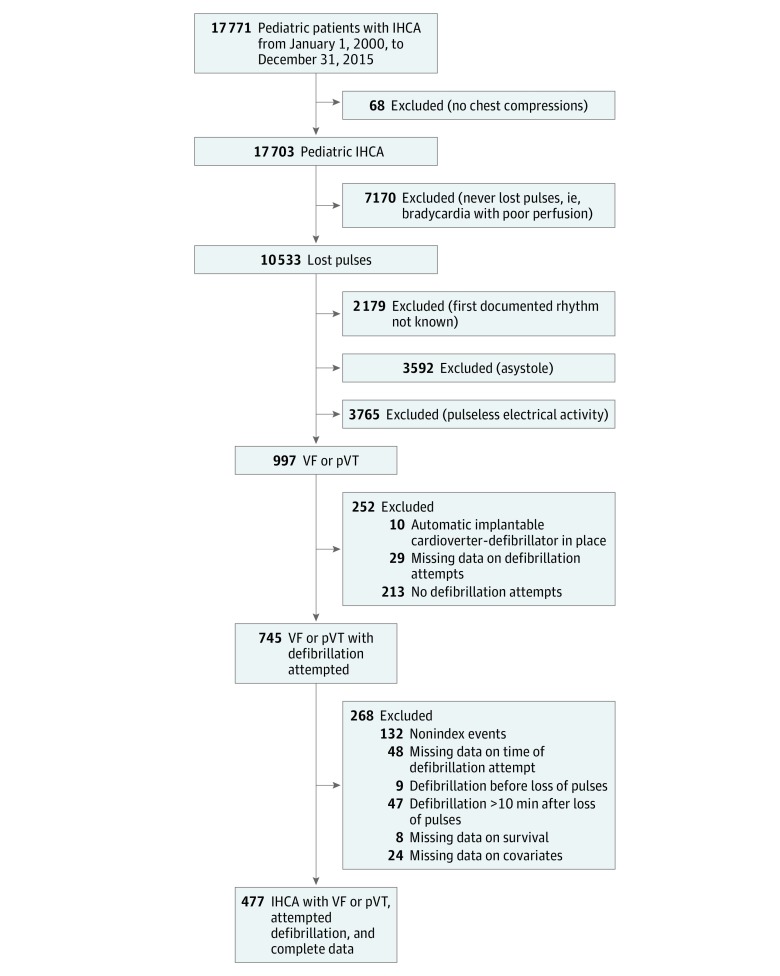
Patient Inclusion and Exclusion Criteria Of 17 771 pediatric IHCA events, 477 were included in the study. IHCA indicates in-hospital cardiac arrest; pVT, pulseless ventricular tachycardia; VF, ventricular fibrillation.

Of 17 771 pediatric patients who experienced cardiac arrest events included in the GWTG-R registry from January 1, 2000, to December 31, 2015, 477 children from 113 hospitals met inclusion criteria and were evaluated in the primary analysis (Figure 1). Patient and event characteristics are provided in Table 1. Among these 477 patients with a pulseless shockable rhythm, the median (IQR) age was 4 years (3 months to 14 years), 285 (60%) were male, 192 (40%) had an initial pulseless rhythm of pVT, and 285 (60%) had VF. Twenty-two percent of the patients (103 of 477) initially received CPR while they still had a pulse, but their initial documented rhythm at the time of pulselessness was a shockable rhythm. Thirty-one percent of patients (147 of 477) had hypotension prior to their pVT/VF arrest. Thus, in this cohort, 44% of patients (210 of 477) were hypotensive and/or receiving CPR for hypoperfusion prior to the development of a fatal arrhythmia.

**Figure 2.**
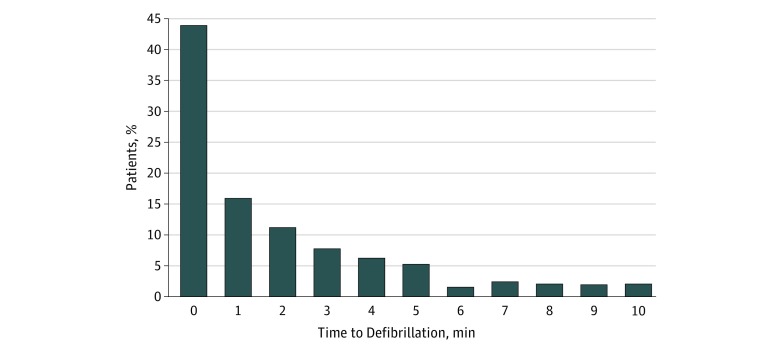
Distribution of Time to First Defibrillation Attempt Proportion of study participants per 1 minute elapsed between loss of pulse and time to first defibrillation attempt.

The median (IQR) time to chest compressions was 0 (0-0) minutes. The median (IQR) time to first defibrillation attempt was 1 minute (1-3 minutes). The distribution of time to first defibrillation attempt is provided in Figure 2, with 71% of all events receiving a shock in 2 minutes or less.

### Survival

**Figure 3.**
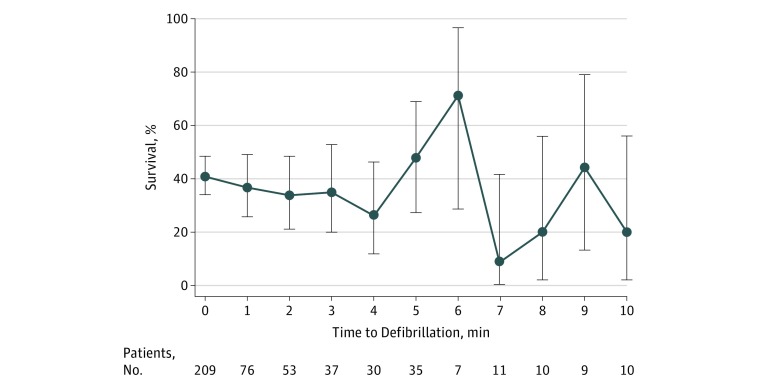
Survival According to Minute of First Defibrillation Attempt Percentage of patients (with 95% confidence intervals [error bars]) who survived to hospital discharge for each 1 minute elapsed between loss of pulse and time to first defibrillation attempt.

Overall, 38% of the patients (179 of 477) survived to hospital discharge, and 23% of the subgroup with CPR prior to pulselessness (24 of 101) survived to hospital discharge. Comparisons of patient and event characteristics between patients who survived to hospital discharge and those who did not survive are provided in Table 1. The median (IQR) time to first defibrillation attempt was 1 minute (0-3 minutes) in both survivors and nonsurvivors. Time to first defibrillation attempt was not associated with survival in unadjusted analysis (RR per minute increase, 0.96; 95% CI, 0.92-1.01; *P* = .15) (Figure 3) or in adjusted analysis (RR, 0.99; 95% CI, 0.94-1.06; *P* = .86). Quadratic and cubic terms of time to first defibrillation attempt were not significant. The final multivariable model for survival is presented in Table 2.

**Table 2.** Multivariable Model for Survival for Time to Defibrillation

Multivariable Model for Survival	RR (95% CI)	*P* Value
Time to defibrillation (per minute)	0.99 (0.94-1.06)	.86
Illness category		
Medical cardiac	0.98 (0.81-1.20)	.87
Medical noncardiac	0.37 (0.24-0.58)	<.01
Newborn	1.51 (1.04-2.21)	.03
Surgical cardiac	1 [Reference]	NA
Surgical noncardiac	0.47 (0.27-0.83)	.009
Preexisting conditions		
Respiratory insufficiency	0.58 (0.45-0.74)	<.001
Renal insufficiency	0.42 (0.21-0.87)	.02
Baseline depression in central nervous system function	0.49 (0.27-0.90)	.02
Major trauma	0.32 (0.14-0.77)	.01
Location		
Critical care area	1 [Reference]	NA
Emergency department	0.72 (0.35-1.50)	.38
Floor with telemetry or step-down unit	1.28 (0.91-1.81)	.15
Floor without telemetry	2.11 (1.21-3.69)	.008
Other	1.35 (1.05-1.72)	.02

Abbreviations: NA, not applicable; RR, risk ratio.

Time to first defibrillation attempt was 2 minutes or less for 338 patients (71%). Children who experienced an IHCA on the wards were less likely to have a time to first defibrillation attempt of 2 minutes or less than those in the intensive care unit (ICU) (48% [11 of 23] vs 72% [268 of 371], *P* = .01). Patient characteristics according to dichotomized defibrillation time are provided in Table 1. There was no difference in survival between those with a time to initial defibrillation attempt of 2 minutes or less compared with those with a time of more than 2 minutes by unadjusted analysis (132 of 338 [39%] vs 47 of 139 [34%]; RR, 0.87; 95% CI, 0.66-1.13; *P* = .29) or adjusted analysis (RR, 0.99; 95% CI, 0.75-1.30; *P* = .93).

### Secondary Outcomes

Return of circulation was achieved in 350 patients (73%). Patient characteristics according to ROC are provided in eTable 1 in the Supplement. Time to first defibrillation attempt was not associated with ROC by unadjusted analysis (RR per minute increase, 0.99; 95% CI, 0.96-1.01; *P* = .28) or by adjusted analysis (RR per minute increase, 0.99; 95% CI, 0.98-1.01; *P* = .42).

Two hundred eighty-four patients (60%) survived at 24 hours after IHCA. Characteristics according to 24-hour survival are provided in eTable 2 in the Supplement. Time to first defibrillation attempt was not associated with 24-hour survival in unadjusted analysis (RR per minute increase, 0.95; 95% CI, 0.81-1.13; *P* = .58) or adjusted analysis (RR per minute increase, 0.99; 95% CI, 0.96-1.01; *P* = .37).

Data on neurologic outcome were missing in 65 patients, corresponding to 14% of all patients and 36% of those who survived to hospital discharge. Of those with available neurologic outcome data, 98 of 412 patients (24%) survived with a favorable neurologic outcome, and 98 of 114 patients who survived to hospital discharge (86%) had a favorable neurologic outcome. Patient and event characteristics according to neurologic outcome are provided in eTable 3 in the Supplement. Time to first defibrillation attempt was not associated with favorable neurologic outcome in unadjusted analysis (RR per minute increase, 0.97; 95% CI, 0.90-1.04; *P* = .38) or adjusted analysis (RR per minute increase, 0.98; 95% CI, 0.90-1.07; *P* = .68). Results were similar when using different definitions of neurological outcomes (eTable 4 in the Supplement).

### Additional Prespecified Sensitivity Analyses

Using multiple imputation for missing data, we were able to increase the evaluable population from 477 patients to 557 patients. For this larger population, time to first defibrillation attempt was not associated with survival by unadjusted analysis (RR per minute increase, 0.96; 95% CI, 0.90-1.02; *P* = .23) or adjusted analysis (RR per minute increase, 1.00; 95% CI, 0.94-1.06; *P* = .94).

In the analysis including subsequent events, time to first defibrillation attempt was not associated with ROC (eAppendix in the Supplement).

### Post Hoc Sensitivity Analyses

Among 253 patients who were not receiving vasopressors, inotropes, or antiarrhythmic drugs at the time of the cardiac arrest, 41% survived to hospital discharge. Time to first defibrillation attempt in these patients was not associated with survival in unadjusted analysis (RR, 0.95; 95% CI, 0.89-1.02; *P* = .17) or in adjusted analysis (RR, 0.97; 95% CI, 0.91-1.04; *P* = .36). When time to first defibrillation attempt was considered as a categorical variable, it was again not associated with survival in unadjusted analysis (RR, 0.76; 95% CI, 0.53-1.08; *P* = .12) or adjusted analysis (RR, 0.85; 95% CI, 0.60-1.21; *P* = .36).

Among the 374 patients who did not receive chest compressions prior to pulselessness from the initial cohort of 477 patients, 41% survived to hospital discharge. In this subgroup, time to first defibrillation attempt was associated with survival in the unadjusted analysis (RR, 0.94; 95% CI, 0.89-0.99; *P* = .03) but not in the adjusted analysis (RR, 0.98; 95% CI, 0.93-1.03; *P* = .34). In the full cohort, results were similar when the variable of chest compressions prior to pulselessness was included in the multivariable model (RR, 0.98; 95% CI, 0.93-1.04).

In a further analysis adding the 24 patients with 10- to 20-minute time from pulselessness to defibrillation attempt to the 477 patients in the primary analyses (ie, 501 patients total), time to first defibrillation attempt was associated with decreased survival in unadjusted analysis with time as a continuous variable (RR, 0.96; 95% CI, 0.93-1.00; *P* = .04) but was not associated with survival in adjusted analysis (RR, 0.98; 95% CI, 0.95-1.01; *P* = .24). When treating time to first defibrillation attempt as categorical (≤2 minutes vs >2 minutes) in this subgroup, time to first defibrillation attempt was not associated with survival in unadjusted analysis (RR, 0.83; 95% CI, 0.63-1.10; *P* = .19) or adjusted analysis (RR, 0.93; 95% CI, 0.72-1.21; *P* = .60).

In addition, we compared the 338 patients with a time to first defibrillation attempt of 2 minutes or less and the 16 patients with a time to first defibrillation attempt of more than 12 minutes. Again, time to first defibrillation attempt was not associated with survival to hospital discharge in unadjusted analysis (RR, 0.48; 95% CI, 0.17-1.34; *P* = .16) or in adjusted analysis (RR, 0.49; 95% CI, 0.20-1.20; *P* = .12). Finally, we conducted further post hoc sensitivity analyses both in relation to location of the cardiac arrest within the hospital and in relation to the size of hospital contributing data with no impact on the results (eAppendix, eTable 5, and eTable 6 in the Supplement).

## Discussion

In this study, we examined a large registry cohort of children with pulseless IHCA, a shockable first documented rhythm, and complete data on important predefined potential confounding factors. Contrary to our hypothesis, we did not find a significant association for time elapsed from loss of pulse to first defibrillation attempt and survival to hospital discharge. Given that the consistency of large animal laboratory models,^36^ adult OHCA,^37^ adult IHCA,^20^ and recent pediatric OHCA data^35^ have established that the time to first defibrillation attempt for pVT and VF is associated with survival, it is important to explore what may be unique about pediatric IHCA and the implications for future research and clinical approach.

Previous animal and clinical data have generally shown that a shorter time to first defibrillation attempt is associated with better outcomes. Many animal studies have shown that delays in first defibrillation attempt are associated with worse outcomes, especially in models with preshock no-flow periods mimicking adult OHCA.^36,37^ Notably, Valenzuela et al^37^ reported a time-dependent dose-response curve for time to first defibrillation attempt and survival among adults with OHCA with a 10% increase in mortality with each minute of delay. They also showed that the effect of time to first defibrillation attempt is diminished by providing CPR. In addition, Chan et al^20^ showed that adults with IHCA in the GWTG-R database were much more likely to survive to hospital discharge when the first defibrillation attempt was provided in 2 minutes or less after pulselessness vs more than 2 minutes after pulselessness. They also demonstrated a dose-response effect that manifested as a decrement in survival to discharge with additional delays in time to first attempted defibrillation. Mitani and colleagues^35^ showed that children with OHCA due to a shockable rhythm had higher rates of 1-month survival and positive neurologic outcomes after bystander-initiated public access defibrillation (mean [SD] time to defibrillation attempt, 3.3 [3.7] minutes) compared with controls who received emergency medical service defibrillation (mean [SD] time to defibrillation attempt, 12.9 [5.8] minutes). They also showed that the time from collapse to defibrillation with an automated external defibrillator was associated with an 8% worsening in 1-month survival for each minute of delay. However, at least 1 clinical study^34^ did not show an association with time to first shock and survival in all populations.

Why did this pediatric IHCA study fail to demonstrate a difference in outcome with time to first defibrillation attempt? The first possibility is that among children who experience an IHCA from a shockable rhythm, there truly is no association between time to first defibrillation attempt and survival to discharge. The highly monitored status, rapid recognition with nearly immediate CPR, and perfusion of myocardium may attenuate the effects of time to first defibrillation attempt. This has been documented in other populations. In a single-site study, Herlitz et al^34^ described a cohort of 254 adult patients with IHCA with a first documented rhythm of VF, in which hospital location of the IHCA was an important effect modifier. Delay in first shock was associated with lower likelihood of survival for patients on unmonitored wards but not for patients on monitored wards. Most pediatric IHCA events occur within a critical care setting, as opposed to adult IHCAs, of which more than 40% occur outside the critical care setting.^38^ Berg et al^38^ reported the proportion of children who experience an IHCA in the ICU setting has increased significantly over time. Only 5% of our cohort with a first documented shockable rhythm (23 of 477) experienced an IHCA on the wards, as opposed to 45% of the adult IHCA cohort (3063 of 6789) described by Chan et al.^20^ When comparing process measures by location in our cohort, children who experienced an IHCA on the wards were less likely to receive a first defibrillation attempt in 2 minutes or less than those in the ICU, but there were very few of them. It is possible that for the highly monitored pediatric or adult patient who experiences a shockable cardiac arrest and has CPR started immediately, time to first defibrillation attempt does not have as strong an association with survival to discharge as it does for those who are on the wards, particularly unmonitored wards.

A second possibility is that the ability to alter IHCA outcome with time to first defibrillation attempt is not demonstrable in the child who is critically ill. Note that nearly one-third (147 of 477 [31%]) of this pediatric cohort had hypotension prior to their pVT or VF arrest and an additional 103 of 477 (22%) received chest compressions prior to becoming pulseless. In this cohort, 44% (210 of 477) of patients were hypotensive and/or receiving CPR for hypoperfusion prior to the development of a fatal arrhythmia. If the myocardial ischemia is not sudden prior to a cardiac arrest, the cellular milieu may be metabolically depleted.^39^ This may be very different from an adult with abrupt coronary artery occlusion or an adolescent with abrupt-onset commotio cordis. Our post hoc sensitivity analyses eliminating patients receiving vasopressors, inotropes, or antiarrhythmic drugs prior to IHCA did not find an association with time to first defibrillation attempt and survival, suggesting that the explanation of metabolic depletion is less likely. However, some patients in this cohort were receiving CPR with a pulse prior to an arrhythmia and therefore did not represent a true sudden arrest either. Moreover, the sample size of patients with true sudden arrest (even if isolated in an analysis) is very small, and whether defibrillation would be beneficial within the population of children with sudden arrest remains unanswered in the current analysis.

Thus, a third possibility is that a true difference in outcome based on time to first defibrillation attempt may exist, but in addition to a potentially inadequate sample size, misclassification could obscure the relationship. A potential source of misclassification could be related to delayed recognition of pVT or VF by hospital staff because it occurs infrequently, thus leading to inaccurate documentation of time of onset of IHCA and time to first defibrillation attempt. This theory is supported by pediatric simulation studies in which there is frequently a delay or complete lack of pVT or VF recognition.^6,21,40,41,42^ Another source of bias could be miscoding of the actual time to defibrillation attempt, which is a risk in any quality improvement database with rewards for meeting guideline targets. Thus, we originally chose to exclude from our primary analysis events in which the time to first defibrillation attempt delivered was reported to be more than 10 minutes, as these may represent error in reporting. Upon adding the extremes into the data set for post hoc sensitivity analysis (ie, those with attempted defibrillation 10-20 minutes after loss of pulse), time to first defibrillation attempt was associated with survival to discharge in unadjusted analysis, but not after adjustment for possible confounders.

In summary, we do not have a single comprehensive explanation of why the association of time to first defibrillation attempt for IHCA due to shockable rhythm and survival would be different in children than adults, or for IHCA vs OHCA. Possible etiologies explored include cardiac physiology varying by chronological age, physiology of critical illness preceding IHCA attenuating the effects of rapid defibrillation, rapidity of recognition of IHCA, impact of high-quality CPR provided in the ICU environment minimizing degradation in cellular milieu, and/or bias of delayed recognition of arrhythmia or inaccurate documentation of time elements. It is also possible that there is a subset of children in whom rapid defibrillation does make a difference, but we do not have the power to distinguish this group.

### Limitations

The results should be interpreted in the context of the study design and some potential limitations. First, despite including patients from multiple hospitals over 15 years, we were limited by the sample size and the fact that most patients had time to first defibrillation attempt within 2 minutes of pulselessness (Figure 2). Although we found no association between time to first defibrillation attempt and outcomes, the confidence intervals for many analyses cannot rule out a clinically meaningful association. Second, cardiac arrest is an acute event in an often chaotic environment, which might have led to some misclassification of the included variables, particularly time.^43,44,45,46,47^ Most likely this potential misclassification is nondifferentiated (ie, not related to outcomes)^46^ and would, therefore, in most instances, bias the results toward the null.^48^ Third, as with any observational study, there might be unmeasured or residual confounding that could influence the findings.

## Conclusions

In this large, multicenter cohort of pediatric IHCA with a first documented shockable rhythm, the median time to first defibrillation attempt was 1 minute, with 71% of events reported as receiving a first defibrillation attempt in 2 minutes or less, and an overall survival to discharge of 38% (179 of 477 patients). Contrary to published adult IHCA and pediatric OHCA data, we did not observe a significant association between time to first defibrillation attempt and survival to hospital discharge.

## Notes

Supplement. **eAppendix.** Supplementary Methods and Results **eReferences** **eTable 1.** Characteristics of the Study Population According to Whether or Not They Achieved Return of Circulation (ROC) **eTable 2.** Characteristics of the Study Population According to 24-Hour Survival **eTable 3.** Characteristics of the Study Population According to Neurological Outcome **eTable 4.** Sensitivity Analyses for Various Definitions of Neurological Outcome **eTable 5.** Time to Defibrillation According to Location Within Hospitals **eTable 6.** Time to Defibrillation and Survival According to Quartiles of Pediatric Cardiac Arrest Cases Contributed
