# Error in Key Points

**DOI:** 10.1001/jamanetworkopen.2018.4644

**Published:** 2018-10-19

In the Original Investigation titled “Association Between Time to Defibrillation and Survival in Pediatric In-Hospital Cardiac Arrest With a First Documented Shockable Rhythm,”^1^ published September 21, 2018, the Findings section of the Key Points should have listed the number of pediatric patients as 477. This article has been corrected.
