# Supplementary material for: Association Between Time to Defibrillation and Survival in Pediatric In-Hospital Cardiac Arrest With a First Documented Shockable Rhythm
Source: JAMA Netw Open. 2018 Sep 21;1(5):e182643. doi: 10.1001/jamanetworkopen.2018.2643 (PMC6324599; doi:10.1001/jamanetworkopen.2018.2643)
Supplement: Supplement. — eAppendix. Supplementary Methods and Results eReferences eTable 1. Characteristics of the Study Population According to Whether or Not They Achieved Return of Circulation (ROC) eTable 2. Characteristics of the Study Population According to 24-Hour Survival eTable 3. Characteristics of the Study Population According to Neurological Outcome eTable 4. Sensitivity Analyses for Various Definitions of Neurological Outcome eTable 5. Time to Defibrillation According to Location Within Hospitals eTable 6. Time to Defibrillation and Survival According to Quartiles of Pediatric Cardiac Arrest Cases Contributed [file jamanetwopen-1-e182643-s001.pdf]

Supplementary Online Content

Hunt EA, Duval-Arnould JM, Bembea MM, et al. Association Between Time to Defibrillation and Survival in Pediatric In-Hospital Cardiac Arrest With a First Documented Shockable Rhythm. *JAMA Netw Open.* 2018;1(5):e182643. doi:10.1001/jamanetworkopen.2018.2643

eAppendix. Supplementary Methods and Results

eReferences

eTable 1. Characteristics of the Study Population According to Whether or Not They Achieved Return of Circulation (ROC)

eTable 2. Characteristics of the Study Population According to 24-Hour Survival

eTable 3. Characteristics of the Study Population According to Neurological Outcome

eTable 4. Sensitivity Analyses for Various Definitions of Neurological Outcome

eTable 5. Time to Defibrillation According to Location Within Hospitals

eTable 6. Time to Defibrillation and Survival According to Quartiles of Pediatric Cardiac Arrest Cases Contributed

This supplementary material has been provided by the authors to give readers additional information about their work.

© 2018 Hunt EA et al. *JAMA Network Open.*

eAppendix – Supplementary Methods and Results

Detailed Methods:

Planned sensitivity analyses for the primary outcome

We performed preplanned sensitivity analyses for the primary outcome using multiple imputation, assuming data was “missing at random”. We imputed values for categorical variables, including survival to hospital discharge, using the fully conditional specification method.[1] A total of 10 data sets were created. Time to first defibrillation attempt was then imputed using a zero-inflated Poisson distribution for each of these 10 datasets.[2] We then performed the main analysis on these 10 data sets and combined the results using SAS “proc mianalyze”.

Planned secondary analysis

As a predefined secondary analysis, we evaluated ROC in a cohort including non-index events. For this analysis, we used a similar approach as described in the main manuscript but also accounted for repeated events within the same patients using GEE.

Results

Pre-specified sensitivity analysis

In the analysis including non-index events, 564 events were included of which 477 (85%) were index events, i.e. the patient’s initial cardiac arrest. In unadjusted analysis, time to first defibrillation attempt was not associated with ROC (RR per minute increase: 0.99 [95%CI: 0.97, 1.01], p = 0.21). In the multivariable analysis, accounting for the same variables as in the main multivariable analysis for ROC and for repeated events

© 2018 Hunt EA et al. *JAMA Network Open.*

within the same patient, time to first defibrillation attempt was also not associated with ROC (RR per minute increase: 0.99 [95%CI: 0.97, 1.01], p = 0.40).

Additional post-hoc sensitivity analyses

The location of cardiac arrests within the hospital was associated time to defibrillation (Table 1) but there was notable overlap in the distributions (eTable 5). Due to concern that possible collinearity may impact the results of the model, post-hoc sensitivity analysis excluding the location variable was performed. When this variable was excluded from the model, the results were similar (RR: 1.00 [95%CI: 0.94, 1.07]). In addition, when patients on the floor ("Floor with telemetry/step-down unit" and "Floor without telemetry") were excluded from the analysis due to very small numbers in those categories, (eTable 5) the results were also similar (RR: 0.99 [95%: 0.93, 1.05]).

Given that 113 hospitals contributed to the 477 patients, there was marked variability in the number of patients per site. When dividing the hospital into quartiles, the quartile contributing the fewest cardiac arrests (≤ 2) had significantly lower survival to discharge (26%) than those contributing more cardiac arrests, but there was no difference in the median (IQR) time to defibrillation between the four quartiles, (eTable 6). In order to explore whether the relationship would be different when analyzing only hospitals that contributed a large volume of cardiac arrests we performed additional analysis. When we restricted the unadjusted analysis to the nine hospitals with the most cases (n= 252), the results were similar (RR: 0.97 [95%CI: 0.91, 1.03)]. Given the low sample size, we are not able to perform adjusted analyses in this subgroup.

© 2018 Hunt EA et al. *JAMA Network Open.*

eReferences

1. van Buuren S. Multiple imputation of discrete and continuous data by fully conditional specification. *Stat Methods Med Res*. 2007;16(3):219-242. doi:10.1177/0962280206074463

2. Pahel BT, Preisser JS, Stearns SC, Rozier RG. Multiple imputation of dental caries data using a zero-inflated Poisson regression model. *J Public Health Dent*. 2011;71(1):71-78. doi:10.1111/j.1752-7325.2010.00197.x

© 2018 Hunt EA et al. *JAMA Network Open.*

eTable 1. Characteristics of the study population according to whether or not they achieved Return of Circulation (ROC)

Characteristics of the Study Population According to ROC	No ROC (n = 127)	ROC (n = 350)	p-value
**Demographics**			
Gender			0.67
Male	78 (61)	207 (59)	
Female	49 (39)	143 (41)	
Age group			0.15
Neonate (< 1 month)	16 (13)	73 (21)	
Infant (1 month up to 1 year)	20 (16)	57 (16)	
Child (1 year to 12 years)	44 (35)	117 (33)	
Adolescent (> 12 years)	47 (37)	103 (29)	
**Illness Category**			< 0.001
Medical cardiac	24 (19)	107 (31)	
Medical non-cardiac	49 (39)	74 (21)	
Newborn	1 (1)	6 (2)	
Surgical cardiac	18 (14)	120 (34)	
Surgical non-cardiac	35 (28)	43 (12)	
**Pre-Existing Conditions**			
Heart failure this admission	23 (12)	67 (19)	0.89
Heart failure prior to this admission	12 (9)	44 (13)	0.42
Myocardial infarction failure this admission	1 (1)	12 (3)	0.20
Myocardial infarction prior to this admission	0 (0)	5 (1)	0.33
Hypotension	45 (35)	102 (29)	0.22
Respiratory insufficiency	67 (53)	169 (48)	0.41
Renal insufficiency	19 (15)	33 (9)	0.10
Hepatic insufficiency	11 (9)	10 (3)	0.01
Metabolic/electrolyte abnormality	17 (13)	65 (19)	0.22
Baseline depression in CNS function	15 (12)	41 (12)	1.00
Acute stroke	2 (2)	2 (1)	0.29
Acute non-stroke CNS event	18 (14)	31 (9)	0.12
Pneumonia	9 (7)	19 (5)	0.51
Septicemia	16 (13)	37 (11)	0.51
Major trauma	33 (26)	27 (8)	< 0.001
Metastatic/hematologic malignancy	4 (3)	7 (2)	0.49
**Location and Time of the Arrest**			
Location			0.005
Critical care area	98 (77)	273 (78)	
Emergency department	17 (13)	18 (5)	
Floor with telemetry/step-down unit	0 (0)	7 (2)	
Floor without telemetry	5 (4)	11 (3)	
Other	7 (6)	41 (12)	
Time of week			0.04
Weekday	87 (69)	272 (78)	
Weekend	40 (31)	78 (22)	
Time of day			0.35
Daytime	89 (70)	261 (75)	
Nighttime	38 (30)	89 (25)	
**Characteristic of the Arrest**			

© 2018 Hunt EA et al. *JAMA Network Open.*

Witnessed	125 (98)	337 (96)	0.37
Monitored	121 (95)	337 (96)	0.60
Mechanical ventilation in place	84 (66)	246 (70)	0.43
**Characteristics of the Study Population According to ROC**	**No ROC (n = 127)**	**ROC (n = 350)**	**p-value**
**Characteristic of the Arrest, cont.**			
Vasopressors in place	56 (44)	150 (43)	0.83
Antiarrhythmic in place	7 (6)	35 (10)	0.15
Initial pulseless rhythm			0.40
Pulseless ventricular tachycardia	47 (37)	145 (41)	
Ventricular fibrillation	80 (63)	205 (59)	
Time to chest compressions (min)	0 (0, 0)	0 (0, 0)	0.60
**Hospital Characteristics**			
Type of hospital			0.002
Primarily adult	82 (65)	169 (48)	
Primarily children	45 (35)	181 (52)	
Teaching status			0.12
Major	85 (67)	267 (76)	
Minor	34 (27)	68 (19)	
Non-teaching	8 (6)	15 (4)	
**Year of the Arrest**			0.07
2000 - 2005	53 (42)	107 (31)	
2006 - 2010	37 (29)	132 (38)	
2011 - 2016	37 (29)	111 (32)	

[a] ROC: return of circulation, CNS: central nervous system

© 2018 Hunt EA et al. *JAMA Network Open.*

eTable 2. Characteristics of the study population according to 24-hour survival[a]

Characteristics of the Study Population	No 24-hour Survival (n = 193)	24-hour Survival (n = 284)	p-value
**Demographics**			
Gender			0.22
Male	122 (63)	163 (57)	
Female	71 (37)	121 (43)	
Age group			0.01
Neonate (< 1 month)	24 (12)	65 (23)	
Infant (1 month up to 1 year)	30 (16)	47 (17)	
Child (1 year to 12 years)	66 (34)	95 (33)	
Adolescent (> 12 years)	73 (38)	77 (27)	
**Illness Category**			< 0.001
Medical cardiac	37 (19)	94 (33)	
Medical non-cardiac	78 (40)	45 (16)	
Newborn	1 (1)	6 (2)	
Surgical cardiac	21 (11)	117 (41)	
Surgical non-cardiac	56 (29)	22 (8)	
**Pre-Existing Conditions**			
Heart failure this admission	30 (16)	60 (21)	0.15
Heart failure prior to this admission	15 (8)	41 (14)	0.03
Myocardial infarction failure this admission	3 (2)	10 (4)	0.26
Myocardial infarction prior to this admission	2 (1)	3 (1)	1.00
Hypotension	74 (38)	73 (26)	0.005
Respiratory insufficiency	108 (56)	128 (45)	0.02
Renal insufficiency	29 (15)	23 (8)	0.02
Hepatic insufficiency	12 (6)	9 (3)	0.12
Metabolic/electrolyte abnormality	36 (19)	46 (16)	0.54
Baseline depression in CNS function	34 (18)	22 (8)	0.001
Acute stroke	3 (2)	1 (0)	0.31
Acute non-stroke CNS event	40 (21)	9 (3)	< 0.001
Pneumonia	15 (8)	13 (5)	0.17
Septicemia	25 (13)	28 (10)	0.30
Major trauma	48 (25)	12 (4)	< 0.001
Metastatic/hematologic malignancy	5 (3)	6 (2)	0.76
**Location and Time of the Arrest**			
Location			< 0.001
Critical care area	151 (78)	220 (77)	
Emergency department	26 (13)	9 (3)	
Floor with telemetry/step-down unit	1 (1)	6 (2)	
Floor without telemetry	7 (4)	9 (3)	
Other	8 (4)	40 (14)	
Time of week			0.19
Weekday	139 (72)	220 (77)	
Weekend	54 (28)	64 (23)	
Time of day			0.07
Daytime	133 (69)	217 (76)	
Nighttime	60 (31)	67 (24)	
Witnessed	186 (96)	276 (97)	0.61
Monitored	185 (96)	273 (96)	1.00

© 2018 Hunt EA et al. *JAMA Network Open.*

Mechanical ventilation in place	140 (73)	190 (67)	0.23
Vasopressors in place	90 (47)	116 (41)	0.22
Antiarrhythmic in place	14 (7)	28 (10)	0.41
**Characteristics of the Study Population**	**No 24-hour Survival (n = 193)**	**24-hour Survival (n = 284)**	**p-value**
Initial pulseless rhythm			0.25
Pulseless ventricular tachycardia	84 (44)	108 (38)	
Ventricular fibrillation	109 (56)	176 (62)	
Time to chest compressions (min)	0 (0, 0)	0 (0, 0)	0.61
**Hospital Characteristics**			
Type of hospital			< 0.001
Primarily adult	126 (65)	125 (44)	
Primarily children	67 (35)	159 (56)	
Teaching status			0.01
Major	129 (67)	223 (79)	
Minor	50 (26)	52 (18)	
Non-teaching	14 (7)	9 (3)	
**Year of the Arrest**			0.002
2000 - 2005	83 (43)	77 (27)	
2006 - 2010	57 (30)	112 (39)	
2011 - 2016	53 (27)	95 (33)	

[a] CNS: central nervous system

© 2018 Hunt EA et al. *JAMA Network Open.*

eTable 3. Characteristics of the study population according to neurological outcome[a]

Characteristics of the Study Population	Poor Neurological Outcome (n = 314)	Good Neurological Outcome (n = 98)	p-value
**Demographics**			
Gender			0.56
Male	191 (61)	56 (57)	
Female	123 (39)	42 (43)	
Age group			0.92
Neonate (< 1 month)	55 (18)	20 (20)	
Infant (1 month up to 1 year)	47 (15)	14 (14)	
Child (1 year to 12 years)	109 (35)	32 (33)	
Adolescent (> 12 years)	103 (33)	32 (33)	
**Illness Category**			< 0.001
Medical cardiac	70 (22)	34 (35)	
Medical non-cardiac	104 (33)	10 (10)	
Newborn	3 (1)	3 (3)	
Surgical cardiac	66 (21)	46 (47)	
Surgical non-cardiac	71 (23)	5 (5)	
**Pre-Existing Conditions**			
Heart failure this admission	53 (17)	26 (27)	0.04
Heart failure prior to this admission	34 (11)	17 (17)	0.11
Myocardial infarction failure this admission	7 (2)	5 (5)	0.17
Myocardial infarction prior to this admission	2 (1)	3 (3)	0.09
Hypotension	110 (35)	28 (29)	0.27
Respiratory insufficiency	183 (58)	37 (38)	< 0.001
Renal insufficiency	47 (15)	4 (4)	0.003
Hepatic insufficiency	20 (6)	1 (1)	0.04
Metabolic/electrolyte abnormality	61 (19)	13 (13)	0.18
Baseline depression in CNS function	49 (16)	3 (3)	< 0.001
Acute stroke	4 (1)	0 (0)	0.58
Acute non-stroke CNS event	46 (15)	2 (2)	< 0.001
Pneumonia	25 (8)	1 (1)	0.009
Septicemia	44 (14)	5 (5)	0.02
Major trauma	56 (18)	3 (3)	< 0.001
Metastatic/hematologic malignancy	9 (3)	2 (2)	1.00
**Location and Time of the Arrest**			
Location			0.02
Critical care area	252 (80)	73 (74)	
Emergency department	30 (10)	4 (4)	
Floor with telemetry/step-down unit	3 (1)	3 (3)	
Floor without telemetry	10 (3)	4 (4)	
Other	19 (6)	14 (14)	
Time of week			0.60
Weekday	230 (73)	75 (77)	
Weekend	84 (27)	23 (23)	
Time of day			0.12
Daytime	22 (70)	77 (79)	
Nighttime	93 (30)	21 (21)	

© 2018 Hunt EA et al. *JAMA Network Open.*

Characteristic of the Arrest			
Witnessed	304 (97)	94 (96)	0.75
Monitored	304 (97)	92 (94)	0.23
Mechanical ventilation in place	228 (73)	60 (61)	0.04
**Characteristics of the Study Population**	**Poor Neurological Outcome (n = 314)**	**Good Neurological Outcome (n = 98)**	**p-value**
**Characteristic of the Arrest, cont.**			
Vasopressors in place	146 (47)	38 (39)	0.20
Antiarrhythmic in place	25 (8)	14 (14)	0.07
Initial pulseless rhythm			0.48
Pulseless ventricular tachycardia	128 (41)	44 (45)	
Ventricular fibrillation	186 (59)	54 (55)	
Time to chest compressions (min)			
**Hospital Characteristics**			
Type of hospital			0.82
Primarily adult	178 (57)	57 (58)	
Primarily children	136 (43)	41 (42)	
Teaching status			0.74
Major	220 (70)	73 (74)	
Minor	77 (25)	21 (21)	
Non-teaching	17 (5)	4 (4)	
**Year of the Arrest**			0.24
2000 - 2005	122 (39)	29 (30)	
2006 - 2010	108 (34)	40 (41)	
2011 - 2016	84 (27)	29 (30)	

[a] CNS: central nervous system

© 2018 Hunt EA et al. *JAMA Network Open.*

eTable 4. Sensitivity analyses for various definitions of neurological outcome[a]

Sensitivity Analyses for Various Definitions of Neurological Outcome[a]					
		Unadjusted		Multivariable	
**Good Neurological Outcome**	**Overall**	**RR (95%CI)[b]**	**p-value**	**RR (95%CI)[b]**	**p-value**
PCPC of 1 or 2, or no increase in PCPC from baseline	105/401 (26%)	0.97 (0.90, 1.04)	0.35	0.98 (0.92, 1.05)	0.62
PCPC of 1, 2, or 3	103/412 (25%)	0.98 (0.92, 1.05)	0.54	0.99 (0.92, 1.07)	0.77
PCPC of 1, 2, or 3, or no increase in PCPC from baseline	108/411 (26%)	0.97 (0.91, 1.04)	0.40	0.98 (0.91, 1.05)	0.58

[a] PCPC = pediatric cerebral performance category

[b] Per minute delay in time to defibrillation

© 2018 Hunt EA et al. *JAMA Network Open.*

eTable 5. Time to defibrillation according to location within hospitals

Location within Hospital	N	Mean	SD	Median	First quartile	Third quartile	Min.	Max.
Critical care area	371	2	3	1	0	3	0	10
Emergency department	35	2	2	1	0	4	0	8
Floor with telemetry/step-down unit	7	2	2	1	0	3	0	7
Floor without telemetry	16	4	3	4	1	7	0	9
Other	48	2	2	1	0	2	0	10

eTable 6. Time to defibrillation and survival according to quartiles of pediatric cardiac arrest cases contributed

Hospital Quartile	N	Time to Defibrillation Median (Q1, Q3)	P value	Survival (%)	P value
1: ≤ 2 cases	100	1 (0, 3)	0.94	26/100 (26)	0.01
2: 3 – 16 cases	125	1 (0, 3)		46/125 (37)	
3: 17 – 37 cases	128	1 (0, 3)		60/128 (47)	
4: ≥ 38 cases	124	1 (0, 3)		47/124 (38)	

© 2018 Hunt EA et al. *JAMA Network Open.*
